# The Complex Management of Mechanical Prosthetic Valve Thrombosis

**DOI:** 10.7759/cureus.41214

**Published:** 2023-06-30

**Authors:** Khalid Saeed Al-Asad, Rand Sabanci, Layan El-khatib, Mohammed Qintar, Christopher Hanson

**Affiliations:** 1 Internal Medicine, Michigan State University, East Lansing, USA; 2 Department of Cardiology, Henry Ford Health System, Detroit, USA; 3 Department of Cardiology, Sparrow Hospital Thoracic and Cardiovascular Institute, Lansing, USA

**Keywords:** therapeutic anticoagulation, therapeutic interventions, multidisciplinary heart team, multimodality cardiac imaging, mechanical prosthetic valve thrombosis

## Abstract

Mechanical prosthetic valve thrombosis (PVT) is a serious condition that is associated with various life-threatening complications. The utilization of multimodality imaging techniques is critical in identifying this etiology. Its management is complex and often requires repeat surgical valve replacements. Our report describes the case of a 48-year-old female who presented with mechanical mitral valve thrombosis in the setting of subtherapeutic anticoagulation. Due to her complex surgical history, nonsurgical therapeutic options were initially pursued for management. Through shared decision-making and after exhaustion of other alternatives, she was maintained on optimized medical therapy and was scheduled for repeat elective surgery. After compliance with medical therapy and close monitoring, she improved significantly, and her underlying pathology completely resolved, eliminating the need for surgery. This report indicates that the management of mechanical prosthetic valve thrombosis should be individualized and emphasizes the importance of involving a multidisciplinary team of medical and surgical professionals to achieve the best clinical outcomes.

## Introduction

Prosthetic valve thrombosis (PVT) is a serious complication of valve replacement that is associated with high morbidity and mortality, especially with mechanical prostheses. Prompt recognition of the condition is crucial, as it necessitates the urgent initiation of therapeutic interventions to restore valve function and prevent complications. The diagnostic approach to PVT depends on clinical evaluation, echocardiography, and other imaging modalities, including computed tomography (CT) and fluoroscopy. Different therapeutic interventions are available for PVT, including optimization of anticoagulation, fibrinolytic therapy, and surgery [1,2]. The choice of the optimal management strategy is usually individualized and considers various factors, such as the clinical presentation, thrombus burden, presence of obstruction, location of the valve, the patient's surgical risk, and the experience and expertise of the managing team. This report describes the complex and successful management of a challenging case of mechanical mitral valve thrombosis in a patient with a complex surgical history.

## Case presentation

A 48-year-old female presented with complaints of dyspnea, orthopnea, and progressively worsening fatigue over the course of two weeks. Her medical history was significant for Ehlers-Danlos syndrome, severe mitral insufficiency that was treated with mitral valve replacement with a bi-leaflet mechanical prosthesis ten years prior to presentation, a chronic interstitial lung disease with recurrent pleural effusions and pneumothoraces that had required bilateral thoracotomies and decortications, and severe autonomic dysfunction that had required permanent pacemaker placement.

Upon evaluation, the patient looked to be in acute distress with increased breathing. Her vital signs showed that her heart rate was 131, her respiratory rate was 27, and her oxygen saturation was low at 85% in ambient air. Her temperature and blood pressure were within normal limits. The physical exam was significant for rales on auscultation of the lung fields bilaterally and for pitting edema in the lower extremities up to the knees. The rest of her physical exam was unremarkable. Initial investigatory work-up showed an elevated level of beta-natriuretic peptide (1553 pg/mL) and intermediately elevated high-sensitivity troponin levels (450 pg/mL) that down-trended shortly after (131 pg/mL). The EKG showed sinus rhythm without signs of active ischemia. Chest radiographs showed cardiomegaly with signs of pulmonary vascular congestion, consistent with the clinical picture of acute congestive heart failure. Further testing revealed an international normalized ratio (INR) of 1.0 in the setting of the patient being noncompliant with her medication regimen, including Warfarin, during the few weeks prior to her presentation.

**Figure 1:**
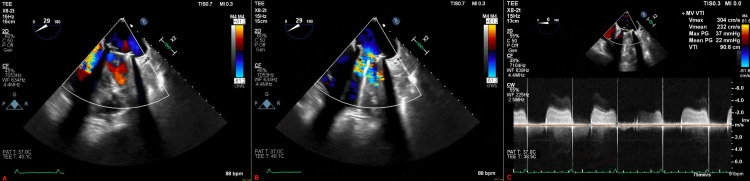
Initial transesophageal echocardiogram. (A) Mechanical prosthesis during systole, (B) mechanical valve prosthesis during diastole with turbulent blood flow on color doppler, and (C) elevated pressure gradients across the mechanical prosthesis.

The patient was admitted to the hospital and started on intravenous (IV) Furosemide as part of the management of a heart failure exacerbation. She was also started on IV Heparin with a plan to bridge to Warfarin therapy given the underlying mechanical mitral prosthesis. Her respiratory status gradually improved throughout the course of her hospital stay, and she was weaned off supplemental oxygen. As part of the complete workup, her transthoracic echocardiogram (TTE) showed normal left ventricular function but revealed right ventricular dysfunction and elevated pressure gradients across the mechanical mitral valve, suggesting severe stenosis. The mitral valve leaflets were not well visualized, and a transesophageal echocardiogram (TEE) was needed to complete the evaluation. TEE revealed a frozen posterior leaflet of the prosthetic valve with an overlying thrombus on the atrial side (Figure 1).

**Figure 2:**
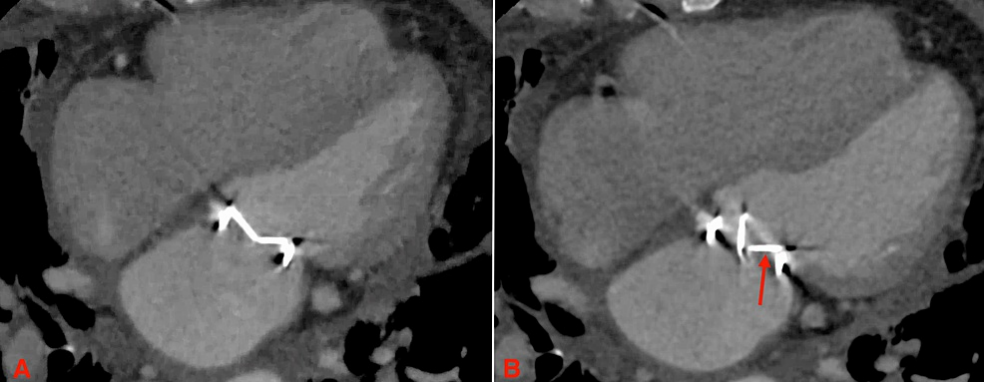
Structural computed tomography of the heart. (A) Appropriately closed mechanical mitral valve leaflets during systole and (B) fixed valve leaflet (red arrow) during diastole with narrowing of the mitral opening.

The patient was counseled on her underlying condition and was informed that the standard of care in her situation would have been mitral valve replacement via conventional surgery. She, however, expressed a desire to explore all other nonsurgical options, considering her prior numerous thoracic surgeries. She was offered fibrinolytic therapy as well as the off-label use of a percutaneous aspiration thrombectomy device, which she opted to proceed with. She was subsequently evaluated by structural cardiology and underwent a structural CT scan of the heart for planning. The study confirmed that the posterior leaflet of the mechanical valve was frozen but did not visualize the thrombus on the mechanical prosthesis (Figure 2). In light of this, the procedure was deemed inappropriate and was subsequently canceled.

**Figure 3:**
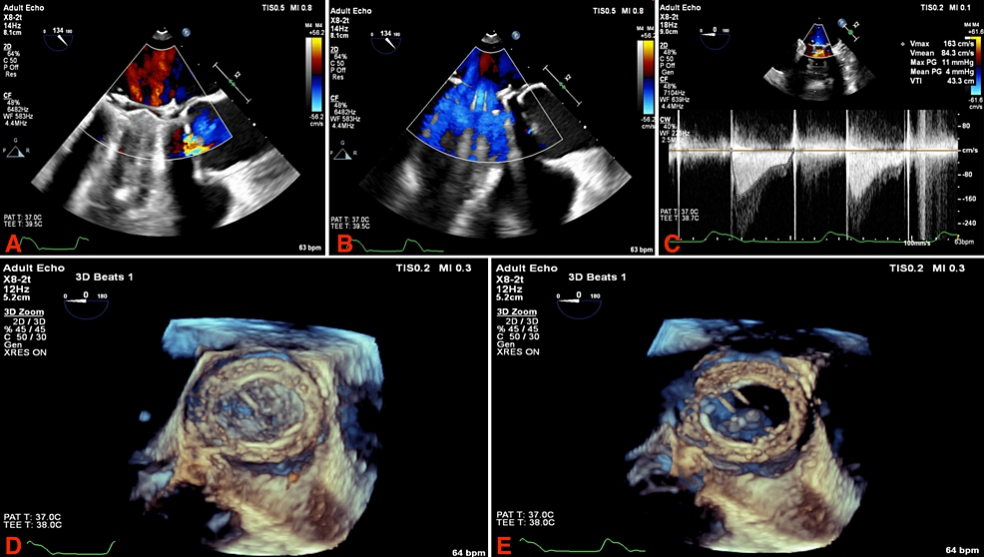
Intra-operative transesophageal echocardiogram. (A) 2D visualization of the mitral prosthesis during systole with color doppler; (B) 2D visualization of the mitral prosthesis during diastole with color doppler; (C) pressure gradients across the mitral prosthesis; (D) 3D visualization of the mitral prosthesis during systole; (E) 3D visualization of the mitral prosthesis during diastole.

Eventually, the patient was evaluated by the cardiothoracic surgical team with the intention of undergoing a repeat surgical mitral valve replacement. By that time, she had significantly improved from a clinical standpoint, and her INR was in the therapeutic range. Through shared decision-making involving the medical and surgical teams as well as the patient, she was discharged from the hospital on Warfarin and oral diuretics and was scheduled for surgery 30 days after her initial presentation. The patient underwent a follow-up TTE 10 days prior to her scheduled surgery with the hope that her frozen leaflet had normalized while her INR was within the goal range; however, the study did not show any significant changes. The patient presented to the operating room on her scheduled appointment, whereby a TEE was performed, showing normalization of the pressure gradients across the mitral valve and that the previously frozen leaflet was then functional (Figure 3). The patient was subsequently discharged and treated medically on an outpatient basis without major complications.

## Discussion

PVT is a pathological entity characterized by thrombus formation on the prosthetic structure and subsequent prosthesis dysfunction. This can be associated with or without thromboembolism. Mechanical prostheses are more durable than bioprosthetic valves and are preferred by younger patients requiring valve replacement to avoid the need for reoperation [3]. Mechanical valves, however, are more thrombogenic and require long-term anticoagulation with a vitamin-K antagonist (VKA) [4]. The annual rate of mechanical valve thrombosis ranges from 0.1% to 5.7%, with the mitral location being roughly five times more commonly involved than the aortic one [2,4]. Mechanical PVT is a complex medical condition and is associated with significant morbidity and mortality. It requires prompt diagnosis and immediate treatment to relieve symptoms and prevent complications.

Mechanical PVT is a subacute to acute process that results in valve dysfunction due to abnormal or absent leaflet motion. Like the case presented in our report, mechanical PVT is typically seen in association with subtherapeutic INR due to inadequate VKA anticoagulation. To date, VKAs remain the cornerstone of therapy for patients with mechanical valve prostheses [4]. Direct oral anticoagulants are not approved for these patients. The use of these agents has been reported to be associated with failure of therapy and the development of PVT [5].

The clinical presentation of mechanical PVT can vary widely and is largely dependent on the acuity of thrombus formation and the degree of valvular obstruction. Obstructive mechanical PVT is associated with hemodynamic compromise and typically presents with the clinical symptoms of reduced cardiac output and heart failure. The presentation is often insidious, with progressively worsening symptoms of dyspnea in most patients. Delays in the diagnosis of obstructive mechanical PVT can lead to the development of fulminant cardiogenic shock. Non-obstructive mechanical PVT is typically present in asymptomatic patients on routine echocardiography or as part of the work-up for the etiology of a thromboembolic event [2]. The patient described in our report had a partial valvular obstruction and presented with symptoms of heart failure. She was diagnosed and started on medical management in a timely fashion before the development of shock.

When suspecting PVT, a thorough physical exam should be performed. One common finding in patients with obstructive PVT is the muffling or disappearance of the prosthetic sounds or the development of a new murmur [1]. Other signs of congestive heart failure, including jugular venous distention, lower extremity pitting edema, and crackles on chest auscultation, are typically present. Initial basic work-up includes cardiac biomarkers, which are typically normal or mildly elevated, and chest radiographs that may show signs of pulmonary vascular congestion and pulmonary edema. A definitive workup incorporates echocardiography, TTE, and TEE, with emphasis on structural abnormalities, leaflet motion, and pressure gradients across the prosthesis. CT or cinefluoroscopic imaging is also part of the investigatory evaluation. Invasive hemodynamic testing is rarely done for the evaluation of PVT [1]. Our patient’s imaging studies revealed elevated pressure gradients across the mitral prosthesis and fixation of one leaflet, and they were able to visualize the thrombus density on echocardiography. Structural CT demonstrated the frozen prosthetic valve leaflet of the mechanical mitral valve.

The current treatment guidelines recommend that patients presenting with a thrombosed mechanical valve undergo definitive therapy. The two options of low-dose continuous-infusion thrombolytic therapy or surgery are both effective, with the decision to proceed with either one based on multiple clinical factors and local experience and expertise [4]. The case described in this report shows that when the decision to proceed with repeat surgical or thrombolytic therapies is difficult to make, medical optimization of anticoagulation and heart failure management can result in positive outcomes and even resolution of valvular obstruction and normalization of prosthesis function. The decision to proceed with this approach, however, is difficult to make and should be individualized based on multiple variables related to the patient's stability and risk factors.

## Conclusions

Mechanical prosthetic valve thrombosis is a complex condition that is associated with life-threatening complications. Choosing the best therapeutic approach for patients with this entity should be individualized based on variable factors. The decision-making process involved is typically complicated and is best rewarded when a multi-disciplinary team of medical and surgical professionals is involved.
